# Prevalence of asymptomatic or “silent” myocardial ischemia in diabetic patients: Protocol for a systematic review and meta-analysis

**DOI:** 10.1371/journal.pone.0252511

**Published:** 2021-06-10

**Authors:** Christophe Dongmo Fokoua-Maxime, Eric Lontchi-Yimagou, Takeude Erwan Cheuffa-Karel, Tchana Loic Tchato-Yann, Simeon Pierre-Choukem

**Affiliations:** 1 University of New York State—University at Albany, School of Public Health, Albany, New York, United States of America; 2 New York State Department of Health, Albany, New York, United States of America; 3 Global Diabetes Institute, Albert Einstein College of Medicine, New York, New York, United States of America; 4 Faculty of Medicine and Biomedical Sciences, University of Yaoundé 1, Yaoundé, Cameroon; 5 Faculty of Medicine and Pharmaceutical Sciences, University of Dschang, Dschang, Cameroon; 6 Health and Human Development (2HD) Research Network, Douala, Cameroon; University of Mississippi Medical Center, UNITED STATES

## Abstract

**Introduction:**

Myocardial ischemia (MI) is a top ranked cause of death among diabetic patients, yet it is mostly asymptomatic or “silent”. There is a need for summary epidemiologic measures on this highly lethal and unnoticeable complication of diabetes. The proposed systematic review and meta-analysis aims to estimate of the global prevalence of silent MI among diabetic patients.

**Methods and analysis:**

This protocol was prepared according to the preferred reporting items for systematic review and meta-analysis protocols (PRISMA-P) statement. The systematic review will include all observational studies published until March 23, 2021 and reporting on the prevalence of silent MI in diabetic patients. Electronic sources including MEDLINE(PubMed), Embase, Cochrane Library, and Web of Science will be searched for potentially eligible studies, restricted to only studies published in English. Two investigators will select studies and use a pre-pilot tested form to extract data. Further, they will independently perform a qualitative assessment of the risk of bias and overall quality of the selected studies, followed by a quantitative assessment using funnel plots and Egger’s tests. The heterogeneity between studies will be assessed with the Cochrane’s Q statistic, and the I^2^ statistic will measure the percentage of variation across studies that is due to their heterogeneity rather than chance; it will decide if a meta-analysis can be conducted. In case a meta-analysis cannot be conducted, a descriptive analysis will be performed. Otherwise, study-specific estimates will be pooled using either a fixed-effects or a random-effects model depending on the value of the I^2^ statistic. Subgroup and random effects meta-regression analyses will be used to further investigate the potential sources of heterogeneity. Finally, sensitivity analyses will be performed to measure the impact of low-quality studies on the results of the meta-analysis, and power calculations will determine the probability that we will detect a true effect if it does exist.

**Strengths and limitations of this study:**

The intended review will provide an up-to-date summary of the global prevalence of silent MI in diabetic patients. We will conduct a thorough literature search for eligible studies, and we will use robust meta-analysis tools to provide reliable estimates of the global prevalence of silent MI in diabetic patients. Two major limitations could be: the predominance of clinical trials that might limit the generalizability of the findings, given that the strict inclusion criteria of these studies might have excluded other patients; the risk of type 1 error emanating from the high number of subgroup and sensitivity analyses.

**PROSPERO registration number:**

CRD42019138136.

## 1. Introduction

Diabetes is the ninth leading cause of death and the fourth leading cause of disability worldwide [1]. This high burden of diabetes is due to its numerous complications, of which cardiovascular diseases (CVD) rank first [2]. The Framingham Heart study was the first to describe the distinctive association between diabetes and myocardial ischemia (MI) [3]. Indeed, diabetic patients have a two- to four-fold higher risk of developing MI than non-diabetic individuals [4, 5], and the risk of a first MI in diabetic patients is equivalent to that of a recurrent MI in non-diabetic patients who had had a previous MI episode [6]. Furthermore, the prognosis after a coronary angioplasty in diabetic patients is shoddier than for non-diabetic individuals [7], and MI claims the outstanding share of 65 to 75 per cent of the deaths among patients with diabetes [8, 9]. Hyperglycemia causes autonomic nerve damages which reduce the capacity of diabetic patients to feel pain [10], making them less able to perceive the discomfort associated with MI; this leads to asymptomatic or “silent” MI [11] which go unnoticed and so are not diagnosed, consequently they do not receive proper care and ultimately result in earlier deaths.

It is estimated that from 2017 to 2045 the adult population of diabetic patients would rise by almost 50 percent [12], which forecasts a future greater global burden of diabetes and silent MI. This urges the need for an up-to-date estimate of the worldwide prevalence of silent MI among diabetic patients to raise awareness about the burden of this highly lethal but clinically imperceptible complication of diabetes. Furthermore, it is important to know the potential factors which contribute to the late or non-diagnosis of silent MI among diabetic patients, as these will provide the foundation for efficient prevention strategies that will save many lives. In 2011, Valensi et al. published a review of the literature on the prevalence, incidence, predictive factors and prognosis of silent MI [13]. The authors did not use the rigorous methods of literature search that are characteristic of a systematic review; therefore, the study might have missed some publications which did assess silent MI in diabetic patients. In 2017, Rados et al. published a meta-analysis of the articles addressing the screening for coronary artery disease in patients with type 2 diabetes [14]. This paper was restricted to clinical trials and thus might not have provided a reliable estimate of the worldwide prevalence of silent MI among diabetic patients.

The proposed systematic review and meta-analysis aims to include all types of studies and will use rigorous methods to provide a reliable estimate of the worldwide prevalence of silent MI among patients with diabetes.

## 2. Objective

To determine the prevalence of asymptomatic or “silent” myocardial ischemia among patients with diabetes.

## 3. Review question

What is the prevalence of asymptomatic or “silent” myocardial ischemia among patients with diabetes?

## 4. Methods

The current protocol has been conceived following the preferred reporting items for systematic review and meta-analysis protocols (PRISMA-P) statement [15]. This protocol was registered in the international prospective register of systematic reviews (PROSPERO) network: registration number CRD42019138136. The anticipated systematic review and meta-analysis will align with this protocol, and will be reported based on the meta-analyses of observational studies (MOOSE) guidelines [16] and the Preferred Reporting Items for Systematic reviews and Meta-Analysis (PRISMA) guidelines [17].

### 4.1. Search methods for the identification of eligible studies

An experienced medical librarian performed a systematic and comprehensive search in the following databases from inception until March 23, 2021: MEDLINE/ PubMed (1947 to March 23, 2021), Embase (1973 to March 23, 2021), Web of Science (1985 to March 23, 2021) and Cochrane Central Register of Controlled Trials (1991 to March 23, 2021). Key terms that were included in the search are: “myocardial infarction”, “silent myocardial ischemia”, “asymptomatic myocardial ischemia”, “unrecognized myocardial infarction”, “asymptomatic coronary artery disease”, “diabetes”, “diabetes mellitus”, “type 2 diabetes mellitus”, “T2DM”, “type 1 diabetes mellitus”, “T1DM”, “maturity onset diabetes of the young (MODY)”, “latent Autoimmune diabetes in adults (LADA)”, “type 3c diabetes’, “steroid-induced diabetes”, “cystic fibrosis diabetes”, “gestational diabetes”, “juvenile diabetes”, and “prevalence”. The search strategy for MEDLINE (via PubMed) is presented in the S1 Appendix. In addition, we will peruse the reference lists of all selected articles to identify potential supplementary data sources.

The search will be repeated prior to the publication of the systematic review in an aim to include any potential eligible study that could have been published since the end of the initial electronic search.

### 4.2. Eligibility criteria for study selection

#### 4.2.1. Study design

The systematic review and meta-analysis will include all cross-sectional, cohort, case-control studies and clinical trials published until March 23, 2021. The search for eligible studies will be supplemented by hand searches in the reference lists of included studies and systematic searches in the gray literature for additional relevant articles.

#### 4.2.2. Participants

Study subjects must be individuals diagnosed with any diabetes phenotype. There will be no restrictions based on sex, age, race/ethnicity, socioeconomic status, or geographic region.

#### 4.2.3. Clinical outcomes

All diabetes cases included in the eligible studies must have been identified by a physician, or diagnosed following a fasting plasma glucose test, and/or an oral glucose tolerance test following the WHO guidelines [18], and/or self-reported. Silent MI must have been diagnosed by a physician and must have been reported in patients who displayed no symptoms during an exercise or pharmaceutical stress test but who exhibited transient ST-segment changes, perfusion defects, or reversible regional wall motion abnormalities [19], or in patients who displayed no symptoms, and a myocardial infarction was later diagnosed based on myocardial imaging evidence or pathological findings on autopsy [20, 21].

#### 4.2.4. Outcome measure

The outcome measure of focus of this review will be the prevalence of silent MI in patients with diabetes.

#### 4.2.5. Language

The search will be restricted to only studies published in English.

#### 4.2.6. Exclusion criteria

We will exclude book chapters, (systematic) reviews and meta-analyses, case-series, fact sheets, white papers, conference proceedings, letters to the editor, commentaries, editorials, and studies without primary data and/or with unachieved methods description. For search leading to similar publications (duplicates), only the most comprehensive report including the largest sample size, a complete methods section and an entire results report will be included. Also, *Kin relationships*, defined as multiple publications describing the same or overlapping series of patients, will be identified; in this instance, only the study with the largest sample size, a comprehensive methods description and a complete results section will be selected.

The complete bibliography of accepted and rejected studies will be available by request to the corresponding author.

### 4.3. Data collection and analysis

#### 4.3.1. Data compilation

Search results will be imported into Endnote. Duplicate articles will be removed, and the remaining references will be alphabetically ordered according to the first authors’ names.

#### 4.3.2. Selection of studies

**Fig 1:**
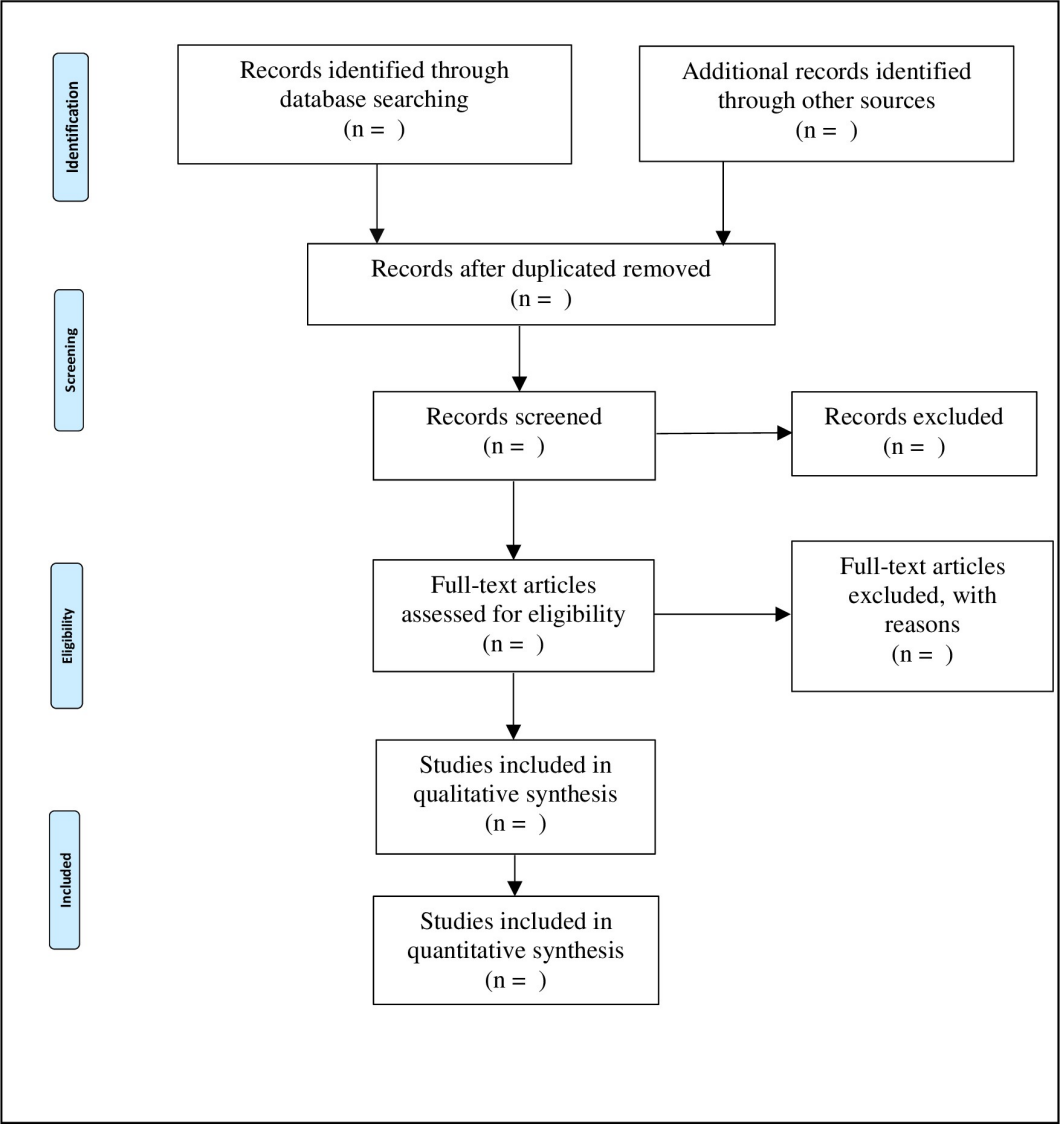
PRISMA flow diagram of the selection of studies to include in a systematic review.

Two authors (CDFM, ELY) will independently screen titles and abstracts record that Will be imported after the literature search. Subsequent to the initial screening, the full texts of records considered eligible will be retrieved and further evaluated for inclusion by the same researchers. Discrepancies in the list of selected articles will be resolved by consensus or by a third reviewer (SPC) if necessary. In line with the PRISMA guidelines [15], a flow diagram will summarize the entire study selection process (Fig 1). The anticipated start date for the selection of articles is March 29, 2021 and the expected completion date is April 9, 2021.

#### 4.3.3. Data extraction and management

Two authors (CDFM, ELY) will use a pre-pilot tested and systematized data extraction form to collect data on:

- Study identification: first author’s name, year of publication, countries and/or regions.
- Study characteristics: study design (cross-sectional, cohort, case-control study or randomized control trial), setting (hospital or community based), duration of follow-up for cohort studies and clinical trials, number of controls per cases and type of matching (if present) for case-control studies.
- Study population: sample size, mean or median age, age range, sex ratio, race/ethnicity distribution.
- Primary exposure: type and sub-type of diabetes.
- Primary outcome: silent MI.
- Covariates: mean and median age at diagnosis of diabetes, duration since the diagnosis of diabetes, type of antidiabetic treatment received (oral antidiabetic drugs, non-insulin injectable antidiabetic drugs, insulin, or a combination of any two or more), proportion of patients with on-target HbA1c levels (<7.0% or study-specific target), proportion of patients with a history of 1 or more acute complications of diabetes (ketoacidosis, hyperosmolar hyperglycemic states, hypoglycemia), proportion of patients with 1 or more chronic complications of diabetes (retinopathy, nephropathy, neuropathy, cerebral artery disease, peripheral vascular disease), method used to detect the silent MI (ECG, ultrasound, cardiovascular magnetic resonance, or autopsy), BMI, proportion of patients with chronic kidney disease (CKD), proportion of patients with pre-existing cardiovascular risk factors (hypertension, dyslipidemia, smoking) or diseases (heart failure, arrythmias, heart valve disease, cardiomyopathies, pericarditis, aortic diseases, stroke), proportion of patients with 1 or more other major comorbidities (human immunodeficiency virus (HIV) infection/ acquired immunodeficiency syndrome (AIDS), cancer, chronic pulmonary disease, chronic liver disease).
- Epidemiological measures: prevalence of silent MI.

We will attempt to contact the authors of articles to obtain important information that could not be found through the retrieval process.

Finally, two other authors (TECK, TLTY) will each randomly select 10 studies and will extract the data one more time to validate that the data extraction process was performed accurately.

#### 4.3.4. Quality assessment and risk of bias of selected studies

Two authors (CDFM, ELY) will separately appraise the methodological quality of each included study, using the tool conceived by Hoy et al for prevalence studies [22]. Each item will be assigned a score of 1 (yes) or 0 (no), and scores will be summed across items to generate an overall quality score that will range from 0 to 10. According to the overall scores, each of the two authors (CDFM, ELY) will classify studies as having a low (>8), moderate (6–8), or high (≤5) risk of bias. Further, the non-weighted Cohen’s kappa statistic will be used to assess the level of agreement between the reviewers. In case of substantial disagreement between the two reviewers (CDFM, ELY), a third reviewer (SPC) will be solicited for arbitration. Following this evaluation, authors of publications containing confusing results and/or results prone to multiple interpretations will be reached out by email to request some clarification or supplemental information. In case a study is excluded, the reasons will be explicitly presented.

If more than 10 eligible studies are found, then publication bias will be visually evaluated by the symmetry of funnel plots, supplemented by a quantitative analysis through an Egger’s test.

#### 4.3.5. Data synthesis

Ad hoc tables will be used to summarize the data of the included studies that are relevant to the specific aims of the prospected systematic review and meta-analysis. The heterogeneity between studies will be measured with the Cochrane’s Q statistic [23]. Further, the authors will use the *I*^*2*^ statistic to estimate the percentage of variation across studies that is due to their heterogeneity rather than chance [24, 25]. The value of the *I*^2^ statistic will be considered small if 0 ≤ *I*^2^ ≤ 25%, medium if 25% < *I*^2^ ≤ 50%, and large if *I*^2^ > 50% [24]. Based on the value of the *I*^*2*^ statistic, the authors will determine whether a meta-analysis is possible. If the *I*^2^ statistic is large, then a meta-analysis will be deemed not possible and a descriptive analysis will be conducted. Otherwise, a meta-analysis will be considered possible, and the *I*^2^ statistic will determine the type of statistical model to be used to pool the study specific estimates. Fixed-effects model analysis will be conducted if the I^2^ statistic is small, otherwise a random-effect model analysis [26] will be performed, after stabilizing the variance of individual studies with the Freeman-Tukey double arc-sine transformation in an aim to minimize the impact that studies with extremely small or extremely large estimates can have on the pooled estimate [24]. R statistical software version 4.0.4 will be used to compute the pooled effect estimates, along with their 95% confidence intervals. The threshold of statistical significance will be a p-value greater than 0.05.

#### 4.3.6. Sources of heterogeneity

The potential sources of heterogeneity will be investigated by subgroup and meta-regression analyses [24]. Subgroup analyses will be performed by type of diabetes, HbA1c levels, sex, race, obesity status, CKD status. If more than 10 studies are included in the quantitative synthesis, then subgroup analyses will be supplemented by random effect meta-regression analyses which will allow the effects of multiple factors (called effect modifiers) to be simultaneously investigated [27]. The potential effect modifiers considered will be the following: sex, race, obesity status, and type of study (observational vs experimental). We will use the model F value and its statistical significance to assess whether there is evidence for an association between any of the covariates and the outcome; all the covariates with p-value <0.2 in bivariable models will be added to the multivariable model, in which a p-value <0.05 will be considered statistically significant. The model fit will be assessed by the adjusted R^2^ which measures the proportion of the between-study variance explained by the covariates [28]. To control for the risk of type I error when performing meta-regression with multiple covariates, we will perform Monte Carlo permutation tests to calculate P values adjusted for type I error and we will check if there is a change in statistical significance [28, 29].

#### 4.3.7. Robustness of the study results

The robustness of the study results will be assessed by performing sensitivity analyses to measure the impact of low-quality studies (identified through their risk of bias). Low-quality studies will be removed one at a time and the meta-analysis will be performed again; we will then compare the results of the meta-analysis with and without the study being assessed, while also accounting for the study sample size, strength of evidence, and impact on aggregated effect size. However, if all the selected studies are at a high risk of bias, we will not conduct sensitivity analyses.

#### 4.3.8. Power analyses

Power analyses will measure the probability that we will detect a true effect if it does exist. Assuming a normal distribution of the effect estimates, the power will be:

Power=1–β, (1)

$$\text{with}\;\beta =\Phi \;\left({\text{C}}_{\alpha }-\lambda \right)\;-\Phi \;\left({–C}_{\alpha }-\lambda \right),$$ (2)

where C_α_ represents the critical value of a *Z*-distribution,

Φ is the standard normal density function obtained through the formula $\Phi =\frac{1}{\sqrt{2\pi }}e{-}^{Z^{2}/2}$,

λ is the true value defined as $\lambda =\frac{1}{\sqrt{vϘ}}$, with Ϙ being the true effect size and *V*_Ϙ_ its variance [30].

Under the assumption that the heterogeneity between the selected studies will be

moderate, *V*_Ϙ_ will be calculated according to the method of Hedges and Pigott: *V*_Ϙ_ = 1.67 x Vy/*k* [31], with *k* being the number of included studies and Vy being the between study variance.

The power calculations will be performed with the *power*.*analysis* function contained in the *dmetar* package of the statistical software R version 4.0.4. Under the aforementioned assumptions, if the true effect is 0.4 and 10 studies are included in the final analyses, then our study will have an 85% power.

#### 4.3.9. Ethics and dissemination

This systematic review was considered exempt from Institutional Review Board approval since our analyses will only include previously published non-identifiable data. The anticipated systematic review and meta-analysis will be reported following the meta-analyses of observational studies (MOOSE) guidelines and the Preferred Reporting Items for Systematic Reviews and Meta-Analyses (PRISMA) statement. The results of the systematic review and meta-analysis will be made available through conference proceedings and peer-reviewed publications.

## 5. Strengths and limitations of this study

The intended review will provide an up-to-date summary of the prevalence of silent MI in diabetic patients. We will conduct a thorough literature search for eligible studies, and we will use robust meta-analysis tools to provide reliable estimates of the prevalence and potential risk factors of silent MI in diabetic patients. Two major limitations could be: the predominance of clinical trials that might limit the generalizability of the findings, given that the strict inclusion criteria of these studies might have excluded other patients; the risk of type 1 error emanating from the high number of subgroup and sensitivity analyses.

## 6. Conclusion

To the best of our knowledge, this systematic review will be the first to estimate the global prevalence of silent MI in patients with diabetes. Despite the high incidence of MI in diabetic patients most of the cases probably go unnoticed because of the diabetes-induced decreased pain sensation; thus, these cases are not medically attended to and subsequently lead to earlier deaths. Therefore, it is important to know the scope of this affection as well as the factors which contribute to the late or non-diagnosis, as these will provide the foundation for efficient prevention strategies that will save many lives. Hence, the intended systematic review and meta-analysis will provide better insights into this highly fatal but clinically imperceptible complication. We believe that this systematic review and meta-analysis will present solid data and robust evidence that will improve clinicians’ practice, trigger future research protocols, and inform health policies aiming to quell the deleterious effects of this highly lethal health hazard.

## Supporting information

S1 Appendix MEDLINE (via PubMed) search strategy. (DOCX)

S1 Checklist PRISMA-P 2015 Checklist. (DOCX)

## References

[1] “Global, regional, and national age-sex-specific mortality for 282 causes of death in 195 countries and territories, 1980–2017: a systematic analysi …—PubMed—NCBI.” https://www.ncbi.nlm.nih.gov/pubmed/30496103 (accessed Apr. 29, 2020). 10.1016/S0140-6736(18)32203-7 PMC6227606 30496103

[2] Tseng C.-H., “Mortality and causes of death in a national sample of diabetic patients in Taiwan,” Diabetes Care, vol. 27, no. 7, pp. 1605–1609, Jul. 2004, doi: 10.2337/diacare.27.7.1605 15220235

[3] Kannel W. B., “Some lessons in cardiovascular epidemiology from Framingham,” Am. J. Cardiol., vol. 37, no. 2, pp. 269–282, Feb. 1976, doi: 10.1016/0002-9149(76)90323-4 1246956

[4] “Excess Mortality among Persons with Type 2 Diabetes | NEJM.” https://www.nejm.org/doi/full/10.1056/NEJMc1515130 (accessed Apr. 29, 2020).

[5] The Emerging Risk Factors Collaboration, “Diabetes mellitus, fasting blood glucose concentration, and risk of vascular disease: a collaborative meta-analysis of 102 prospective studies,” Lancet, vol. 375, no. 9733, pp. 2215–2222, Jun. 2010, doi: 10.1016/S0140-6736(10)60484-9 20609967 PMC2904878

[6] Haffner S. M., Lehto S., Rönnemaa T., Pyörälä K., and Laakso M., “Mortality from coronary heart disease in subjects with type 2 diabetes and in nondiabetic subjects with and without prior myocardial infarction,” N Engl J Med, vol. 339, no. 4, pp. 229–234, Jul. 1998, doi: 10.1056/NEJM199807233390404 9673301

[7] Mori Brooks Maria et al., “Predictors of Mortality and Mortality From Cardiac Causes in the Bypass Angioplasty Revascularization Investigation (BARI) Randomized Trial and Registry,” Circulation, vol. 101, no. 23, pp. 2682–2689, Jun. 2000, doi: 10.1161/01.cir.101.23.2682 10851204

[8] “Cause-specific mortality in a population-based study of diabetes.—PubMed—NCBI.” https://www.ncbi.nlm.nih.gov/pubmed/1951827 (accessed Apr. 29, 2020).

[9] “The Epidemiology of Diabetes Mellitus, 2nd Edition | Wiley,” Wiley.com. https://www.wiley.com/en-us/The+Epidemiology+of+Diabetes+Mellitus%2C+2nd+Edition-p-9780470017272 (accessed Apr. 29, 2020).

[10] Boulton A. J., “Lowering the risk of neuropathy, foot ulcers and amputations,” Diabet. Med., vol. 15 Suppl 4, pp. S57–59, 1998, doi: 10.1002/(sici)1096-9136(1998120)15:4+<s57::aid-dia741>3.3.co;2-4 9868994

[11] Bravo P. E., Psaty B. M., Di Carli M. F., and Branch K. R., “Identification of coronary heart disease in asymptomatic individuals with diabetes mellitus: to screen or not to screen,” Colomb. Med., vol. 46, no. 1, pp. 41–46, Mar. 2015. 26019384 PMC4437286

[12] “IDF Atlas 9th edition and other resources.” https://www.diabetesatlas.org/en/resources/?gclid=Cj0KCQjwy6T1BRDXARIsAIqCTXpGlzUYrak0ExBMo5qjcxj8t1nzxaLeeYNLQMYpcDRxp6Sd5Bg9dbIaAhVbEALw_wcB (accessed Apr. 29, 2020).

[13] Valensi P., Lorgis L., and Cottin Y., “Prevalence, incidence, predictive factors and prognosis of silent myocardial infarction: a review of the literature,” Arch Cardiovasc Dis, vol. 104, no. 3, pp. 178–188, Mar. 2011, doi: 10.1016/j.acvd.2010.11.013 21497307

[14] “Screening for coronary artery disease in patients with type 2 diabetes: a meta-analysis and trial sequential analysis | BMJ Open.” https://bmjopen.bmj.com/content/7/5/e015089 (accessed Apr. 29, 2020). 10.1136/bmjopen-2016-015089 PMC5623378 28490559

[15] Shamseer L. et al., “Preferred reporting items for systematic review and meta-analysis protocols (PRISMA-P) 2015: elaboration and explanation,” BMJ, vol. 349, no. jan02 1, pp. g7647–g7647, Jan. 2015, doi: 10.1136/bmj.g7647 25555855

[16] Stroup D. F. et al., “Meta-analysis of observational studies in epidemiology: a proposal for reporting. Meta-analysis Of Observational Studies in Epidemiology (MOOSE) group,” JAMA, vol. 283, no. 15, pp. 2008–2012, Apr. 2000. doi: 10.1001/jama.283.15.2008 10789670

[17] Liberati A. et al., “The PRISMA statement for reporting systematic reviews and meta-analyses of studies that evaluate healthcare interventions: explanation and elaboration,” BMJ, vol. 339, Jul. 2009, doi: 10.1136/bmj.b2700 19622552 PMC2714672

[18] World Health Organization and International Diabetes Federation, Definition and diagnosis of diabetes mellitus and intermediate hyperglycaemia: report of a WHO/IDF consultation. 2006. Accessed: May 16, 2020. [Online]. Available: http://www.who.int/diabetes/publications/diagnosis_diabetes2006/en/

[19] Conti C. R., Bavry A. A., and Petersen J. W., “Silent Ischemia: Clinical Relevance,” Journal of the American College of Cardiology, vol. 59, no. 5, pp. 435–441, Jan. 2012, doi: 10.1016/j.jacc.2011.07.050 22281245

[20] Turkbey E. B. et al., “Prevalence and Correlates of Myocardial Scar in a US Cohort,” JAMA, vol. 314, no. 18, Art. no. 18, Nov. 2015, doi: 10.1001/jama.2015.14849 26547466 PMC4774246

[21] de Torbal A. et al., “Incidence of recognized and unrecognized myocardial infarction in men and women aged 55 and older: the Rotterdam Study,” Eur. Heart J., vol. 27, no. 6, Art. no. 6, Mar. 2006, doi: 10.1093/eurheartj/ehi707 16478749

[22] Hoy D. et al., “Assessing risk of bias in prevalence studies: modification of an existing tool and evidence of interrater agreement,” J Clin Epidemiol, vol. 65, no. 9, pp. 934–939, Sep. 2012, doi: 10.1016/j.jclinepi.2011.11.014 22742910

[23] Huedo-Medina T. B., Sánchez-Meca J., Marín-Martínez F., and Botella J., “Assessing heterogeneity in meta-analysis: Q statistic or I2 index?,” Psychol Methods, vol. 11, no. 2, pp. 193–206, Jun. 2006, doi: 10.1037/1082-989X.11.2.193 16784338

[24] Higgins J. P. T. and Thompson S. G., “Quantifying heterogeneity in a meta-analysis,” Stat Med, vol. 21, no. 11, pp. 1539–1558, Jun. 2002, doi: 10.1002/sim.1186 12111919

[25] Higgins J. P. T., Thompson S. G., Deeks J. J., and Altman D. G., “Measuring inconsistency in meta-analyses,” BMJ, vol. 327, no. 7414, Art. no. 7414, Sep. 2003. doi: 10.1136/bmj.327.7414.557 12958120 PMC192859

[26] DerSimonian R. and Laird N., “Meta-analysis in clinical trials,” Control Clin Trials, vol. 7, no. 3, pp. 177–188, Sep. 1986. doi: 10.1016/0197-2456(86)90046-2 3802833

[27] Thompson S. G. and Sharp S. J., “Explaining heterogeneity in meta-analysis: a comparison of methods,” Stat Med, vol. 18, no. 20, pp. 2693–2708, Oct. 1999, doi: 10.1002/(sici)1097-0258(19991030)18:20<2693::aid-sim235>3.0.co;2-v 10521860

[28] Kelley G. A. and Kelley K. S., “Statistical models for meta-analysis: A brief tutorial,” World J Methodol, vol. 2, no. 4, pp. 27–32, Aug. 2012, doi: 10.5662/wjm.v2.i4.27 25237614 PMC4145560

[29] “Controlling the Risk of Spurious Findings From Meta-Regression—PubMed.” https://pubmed.ncbi.nlm.nih.gov/15160401/ (accessed Jun. 29, 2020). 10.1002/sim.1752 15160401

[30] M. H. M.Sc.1, P. D. P. Cuijpers2, P. D. T. A. Furukawa3, and A. P. D. D. D. Ebert2, Doing Meta-Analysis in R. Accessed: Mar. 17, 2021. [Online]. Available: https://bookdown.org/MathiasHarrer/Doing_Meta_Analysis_in_R/

[31] Hedges L. V. and Pigott T. D., “The power of statistical tests for moderators in meta-analysis,” Psychol Methods, vol. 9, no. 4, pp. 426–445, Dec. 2004, doi: 10.1037/1082-989X.9.4.426 15598097

