# Correction: Prevalence of asymptomatic or “silent” myocardial ischemia in diabetic patients: Protocol for a systematic review and meta-analysis

**DOI:** 10.1371/journal.pone.0314301

**Published:** 2024-11-18

**Authors:** Christophe Dongmo Fokoua-Maxime, Eric Lontchi-Yimagou, Takeude Erwan Cheuffa-Karel, Tchana Loic Tchato-Yann, Simeon Pierre Choukem

The fifth author’s name is spelled incorrectly. The correct name is: Simeon Pierre Choukem. The correct citation is: Fokoua-Maxime CD, Lontchi-Yimagou E, Cheuffa-Karel TE, Tchato-Yann TL, Pierre Choukem SP (2021) Prevalence of asymptomatic or “silent” myocardial ischemia in diabetic patients: Protocol for a systematic review and meta-analysis. PLOS ONE 16(6): e0252511. https://doi.org/10.1371/journal.pone.0252511
