# Supplementary material for: Prevalence of asymptomatic or “silent” myocardial ischemia in diabetic patients: Protocol for a systematic review and meta-analysis
Source: PLoS One. 2021 Jun 10;16(6):e0252511. doi: 10.1371/journal.pone.0252511 (PMC8191872; doi:10.1371/journal.pone.0252511)
Supplement: S1 Appendix — (DOCX) [file pone.0252511.s001.docx]

S1 Appendix: **MEDLINE (via PubMed) search strategy**

((("Diabetes Mellitus"[MeSH Terms] OR "diabet*"[Title/Abstract]) AND ("myocardial infarction"[MeSH Terms] OR (("silent"[Title/Abstract] OR "asymptomatic"[Title/Abstract] OR "unrecognized"[Title/Abstract] OR "unsuspected"[Title/Abstract]) AND ("ischemia"[Title/Abstract] OR "ischemic"[Title/Abstract] OR "myocardial"[Title/Abstract]))) AND ("prevalence"[MeSH Terms] OR "risk assessment"[MeSH Terms] OR "incidence"[MeSH Terms] OR ("prevalen*"[Title/Abstract] OR "incidence"[Title/Abstract] OR "risk"[Title/Abstract]))) NOT ("Animals"[MeSH Terms] NOT ("Animals"[MeSH Terms] AND "Humans"[MeSH Terms]))) AND "English"[Language] AND ("randomized controlled trial"[Publication Type] OR "controlled clinical trial"[Publication Type] OR "clinical trials as topic"[MeSH Terms] OR "random allocation"[MeSH Terms] OR "double-blind method"[MeSH Terms] OR "single-blind method"[MeSH Terms] OR "clinical trial"[Publication Type] OR "research design"[MeSH Terms:noexp] OR "comparative study"[Publication Type] OR "evaluation studies"[Publication Type] OR "follow-up studies"[MeSH Terms] OR "cohort"[Text Word] OR "prospective studies"[MeSH Terms] OR "cross-over studies"[MeSH Terms] OR "clinical trial"[Text Word] OR (("singl*"[Text Word] OR "doubl*"[Text Word] OR "trebl*"[Text Word]) AND ("mask*"[Text Word] OR "blind*"[Text Word])) OR "placebo*"[Text Word] OR "random*"[Text Word] OR "control"[Text Word] OR "controls"[Text Word] OR "prospectiv*"[Text Word] OR "volunteer*"[Text Word])
